# Who Can Afford Health Care? Evaluating the Socio-Economic Conditions and the Ability to Contribute to Health Care in a Post-Conflict Area in DR Congo

**DOI:** 10.1371/journal.pone.0077382

**Published:** 2013-10-24

**Authors:** Sibylle Gerstl, Justin Sauter, Joseph Kasanda, Alfred Kinzelbach

**Affiliations:** 1 Malteser International, Cologne, Germany; 2 Malteser International, Ariwara, Aru territory, P.O., Democratic Republic of the Congo; 3 Malteser International, Bbunga, Kampala, Uganda; University of Florida, United States of America

## Abstract

**Introduction:**

The Democratic Republic of the Congo is today one of the poorest countries in the world; the health status of the population ranks among the worst in Sub-Saharan Africa. Public health services charge user fees and drug prices. Since 2008, north-eastern Congo is facing a guerrilla war. Malteser International is assisting with free health care for internally displaced persons as well as the general population. Before the incursion the health system was based on user fees. The aim of this study was to determine the socio-economic conditions of the population and to assess their ability to contribute to health care.

**Methodology:**

Heads of 552 randomly selected households in 23 clusters in two health zones were interviewed using a standardised questionnaire.

**Findings:**

The demographic description and socio-economic conditions of the study population were homogenous. Major source of income was agriculture (57%); 47% of the households earned less than US$ 5.5/week. Ninety-two percent of the interviewed households estimated that they would be able to contribute to consultation fees (maximum amount of US$ 0.27) and 79% to the drug prices (maximum amount of US$ 1.10). Six percent opted for free consultations and 19% for free drugs.

**Conclusions:**

Living conditions were very basic; the estimated income of the study population was low. Almost half of the population perceived their current living situation as fairly good/good. More than 90% of the study population estimated to be able to contribute to consultation fees and 80% to drug prices. As a result Malteser International suggested introducing flat-rates for health care services. Once the project ends, the population will have to pay again for their health service. One solution would be the introduction of a health care financing system with the goal to reach universal coverage to health care.

## Introduction

The Democratic Republic of the Congo (DRC) has seen bad governance, wars and armed clashes since independence. From 1996, DRC has been torn by military conflicts characterized by extreme violence, which are still dragging on [1]. As a consequence millions of civilians died, and millions were forced to leave their homes. In 2009, for the 11th consecutive year, DRC was featured on a list of the Top Ten Humanitarian Crises [2]. Since 2008, the north-eastern regions in Province Orientale, which lies in the northeast of the country, have been facing a guerrilla war of the Lord’s Resistance Army (LRA). The LRA has killed more than 2,300 people and abducted more than 3,000 into slavery or forced recruitment. Over 400,000 people have fled their homes for fear of attack, 260,000 of them within DRC [3,4,5]. New figures from 2010 show that over the last two years the LRA has become the most deadly militia in DRC [6,7].

DRC is a resource-rich country including diamonds, gold, uranium, zinc, copper, coltan and tropical forests. Yet mismanagement, corruption and conflict have crippled the formal economy and DRC is one of the poorest countries in the world [8]. It is currently rated number 187 out of 187 countries on the human development index, ranking national achievements in health, education and income [9]. Of the estimated 66 million people in the country, 59% are living with less than US$ 1.25 per day [9,10].

The DRC health system is decentralized, with primary and first referral services integrated in “aires de santé”, so called health zones, that covers an average population of 110,000. A health zone in DRC is equivalent to the district health level in most other countries. Currently, there are 515 health zones in the country [11,12]. Already in the late 1970s the government started to withdraw from financing the health sector, leaving households with the main burden of financing services. Because of the continuing lack of government financing over the last decades, the health zones and facilities operate with considerable autonomy and many facilities became de facto privatized. At present, health personnel in public facilities charge user fees and drug prices in order to finance their remuneration, which can be devastating to the poor and impede access to the most basic health services. The amount of user fees and drug prices is autonomously defined by each single health facility, no standard public sector health fees are existing [11,13,14].

In 2010 in DRC total expenditures on health per capita were US$ 27 and the per capita governmental expenditures on health US$ 6.7 [15]. Another study revealed that households spent on average annually US$ 7 per person in the household for health services [16]. The Congolese Government devoted 1.2% of its gross domestic product on public expenditures on health [15]. The DRC health system is relatively unique in Sub-Saharan Africa in the extent to which it is decentralized and virtually privatized [8].

In consequence the health status of the Congolese people ranks among the worst in Sub-Saharan Africa [8]. According to a study carried out by GAVI in 2009, DRC’s overall health situation has deteriorated to the point that mortality and life expectancy levels are back to a rate last experienced in the 1950s and 1960s [12]. Demographic and health data are generally very low with a life expectancy at birth of 49 years. The country has one of the highest maternal and infant mortality rates in the world. Its maternal mortality rate was estimated at 540 deaths per 100,000 live births and the under-five mortality rate of 170 deaths per 1,000 live births [9,10].

Since 2000 Malteser International (MI) is present in Province Orientale. Even before the LRA incursion, this province was among the least developed parts of the country. Basic infrastructure such as road networks, electricity supply, communication network coverage, access to clean drinking water and especially essential services such as health centres and hospitals are poor. Moreover, there is very limited governmental presence and hardly any functioning judicial system [17].

Since 2009 and as a consequence of the LRA attacks MI has been providing free health care for internally displaced people and the population who had stayed in place. Since 2010 the European Commission's Humanitarian Aid and Civil Protection Department (ECHO) is funding via MI free health care in public health facilities in the health zones of Aba and Faradje, two of the 83 health zones in Province Orientale close to the South Sudanese border. Given the actual state of the health care system in the DRC it is evident that free access to health care is not sustainable. As soon as donors withdraw funding support, the population will have to cover again all expenditures for health care.

Various attempts have been made to assess the socio-economic status and therefore the poverty level of a population. There is little agreement on the best way to approach to measuring socio-economic conditions [18,19,20]. Commonly used indicators of poverty are income and expenditures. However, calculating consumption and particularly income from the raw data is not easy, and the difficulties are discussed extensively in literature [21,22,23,24]. Therefore, adding a set of easily measurable objective indicators but also subjective indicators to the income and expenditures approach improves the explanatory power [25,26,27].

In countries where people are expected to contribute to or to completely pay the cost of health care out of their own pockets people’s ability to pay for health care or the affordability of health care has become an important issue. Research has focused on willingness to pay for essential services and has tended to assume that willingness to pay is synonymous to ability to pay. Households, however, may persist in paying for care, but for mobilizing resources they may sacrifice other basic needs such as food and education, with serious consequences for the household or individuals within it. The opportunity costs of payment make the payment 'unaffordable' because other basic needs are sacrificed [28]. A study carried out in Tanzania confirmed that people may indeed be willing, but may nevertheless not be able to pay for health care [29]. Studies showed that willingness to pay could be influenced by several factors, one of them being the ability to pay, and that willingness to pay for health care is often beyond the financial capacity of a person and depends on multiple factors [30].

Willingness to pay for health services in developing countries is often measured through contingent valuation methods and estimates the value people put in health services. Studying the willingness to pay is increasingly used in proposed community-based health insurance schemes [31,32,33]. For our study setting we found it more suitable to assess the ability to pay for health services and the price range people are able to contribute to health care.

So far no attempts have been made to measure socio-economic conditions of the population in the two health zones as important determinants of health. The aim of this study was to determine the actual socio-economic conditions of the population and to assess their ability to contribute to health care. This information may help both MI and other potential actors in the field of health to better decide if partial or complete participation in user fees for health care should be introduced.

## Methodology

### Ethics statement

There is no nation-wide ethical committee taking care and approving all studies that are carried out in the country. The senior district medical officer of the health district as Ministry of Health representative with his management team is authorized to decide on whether a study may be carried out in the district or not. Thus the district authority takes the role of an ethics committee. Dr. Raymond Kulidri as managing senior medical officer of the health district of Haut Uélé Est (Oriental Province) granted ethical approval prior to the commencement of the study.

Households were only interviewed after written informed consent was given by the head of the household. Everyone was offered the opportunity to refuse participation at any time during the study without any consequences. None of the information gathered was linked to the households or individual interviewees.

As to confidentiality, all data remained anonymous throughout data entry and analysis. Nominal data were not distributed outside the study location, or appear in any report or publication.

### Study design

We used a three-stage cluster sampling method with a probability proportional to the estimated population adapted from the method recommended by the World Health Organization [34]. Randomly selected households were interviewed about their socio-economic conditions and their ability to contribute to health care.

### Study area and population

The study area was Aba and Faradje health zones in Province Orientale in DRC. Aba health zones has approximately 200 villages with a total population of 102,000, Faradje health zone has approximately 158 villages with a total population of 86,000. The study population included everyone already living in the study area during the wartime or being displaced but having returned to the study area for more than three months. The base for the population and village estimation was the latest population census carried out by the Ministry of Health of DRC in 2003 and extrapolated with an annual growth rate of 3% [35]. Houses are usually widely scattered around the village centre and accessible only on foot.

### Sample size

The average household comprises between six and seven members (MI internal report 2010). We took six household members as the conservative average, so with an estimated population size of 188,000 there were about 31,300 households in the study area. Our hypothesis was that 75% of the population are able to contribute to health care. With a precision of 5%, an α-error of 5%, and a design effect of 2, a minimum of 575 households was required. Taking into account the risk that 5% of the households’ information is incomplete 600 households were to be included in the study. We arbitrarily opted for 25 clusters of 24 households as we were expecting a rather homogenous population in our catchment area (small design effect).

### Sampling procedure

A three-stage cluster sampling methodology was used. In the first stage, 25 clusters were randomly selected from a list of both health zones using a probability of allocation proportional to the respective population size of each health zone (14 in Aba and 11 in Faradje health zone). In the second stage, the selected number of clusters per health zones was allocated to villages within this health zone by systematic sampling. The probability of allocation was proportional to the respective population size of each village. In the third stage, 24 households (defined as a group of people being under the responsibility of one person or head of household, regularly sleeping under the same roof and eating together) were randomly selected within a village. A pen was dropped on the ground in the centre of the village, and a line drawn in the direction its tip pointed, towards the edge of the village. Households were counted along this line by walking to the edge of the village. With the use of a random number chosen from a random number table one of these households was selected as the first to be interviewed. The next closest household was then interviewed until 24 households had been included.

If all households of a selected village had been included in the study before completing the needed number of households, the cluster would have been continued by selecting the (geographically) closest village. There again the same standard methodology would have been used to select the first household in the village. As all selected clusters had more than 24 households in the village selected first, there was no need to apply this rule.

### Data collection

Heads of households (defined as household members who were responsible for their households, could therefore give accurate information on the questions asked and were present at the time of the survey) were interviewed at their homes either in the local language Lingala or French using a standardised, semi-closed, pre-piloted questionnaire. The study was anonymous. Four teams of two local interviewers completed one cluster in one day. Prior to initiating the study, the teams received a two days’ on-site training with special focus on interviewing techniques to avoid interviewer bias, including a half-day’s pilot study. The principal study investigator carried out the training and supervised the study teams during the entire time in the field.

### Questionnaire design

The questionnaire consisted of 32 questions in following sections: demographic status of the household, socio-economic conditions and health seeking behaviour.

Socio-economic conditions were measured by asking about the level of lodging, type of latrine, drinking water source, fuel for cooking, means of transportation, possession of agricultural property, possession of animals such as chickens, sheep, pigs and cows, and possession of assets (radio, bed, bicycle). 

For better validation of the answers regarding the income we used two independent methods to collect household income. In the first method we asked about the number of household members in the household who were having a paid work. In a second step the head of household gave us the estimated weekly income of each of these household members. Thirdly, we calculated the weekly income of the households by adding all incomes of the different household members. In the second method, we proposed five different weekly income classes. The heads of the households had to estimate in which weekly income class their households would fall. When comparing the two methods to calculate the households’ weekly income, no significant difference could be seen. Therefore in the results part we refer to the weekly income calculated as the sum of all incomes of all household members. 

To assess expenditures we asked for the major expenditure the household had faced during the three past months prior to the study.

To raise explanatory power we further asked for the main problem the household was struggling with at the time of the study. We also asked for the self-perceived current living situation in the households predefining following four answers: good, fairly good, fairly bad and bad.

To get a better idea about the population’s ability to contribute to health care we asked the heads of the households to give us their estimation of the maximum amount of user fees for health care their households would still be able contributing in the case user fees will again be introduced in the health zones. As in DRC invoicing in the health centres is usually split into consultation fees and costs for drugs, we asked for the contribution to health care separately with two different questions.

To estimate the health seeking behaviour in the study population we asked if a household member has been sick since January 1, 2011. If this was the case we then asked questions about the health seeking behaviour of the last person being sick in the household.

### Data management, analysis and data availability

Data were entered into EpiData 3.0 software (The EpiData Association, Odense, Denmark). Data cleaning checked for inconsistencies in data entry and responses. Data analysis used STATA 8.1 (StataCorp, College Station, TX, USA) and SPSS 11.0 (SPSS. Chicago, USA).

All indicators were calculated as proportions and when appropriate with 95% confidence intervals (95% CI). Estimates of actual design (cluster) effect were also calculated. For comparisons of means for the maximum amount of consultation fees and costs for drugs the independent samples t-test was used. For all variables the design effect was close to 1, therefore we did not report the estimates.

All data that underlie these results were shared electronically and as a hard-copy written in French with all people concerned in DRC and with ECHO in Brussels.

## Results

### Demographic description

Between 14 and 19 February 2011, 552 households (336 in Aba and 216 in Faradje health zones) in 23 clusters were visited at their homes and the heads of households interviewed. For security reasons we were not able to visit two clusters randomly selected prior to the study in Faradje health zone during the field work. Due to the very short notice of the insecure situation (attack on Faradje town two days before the field work) they could not be replaced. There was no difference in the demographic description of the study population both within the two different health zones and between the two health zones. General characteristics of the study population, the households and the heads of households are shown in Table 1.

**Table 1:** General characteristic of the study population and the heads of households interviewed in Aba and Faradje health zones, Province Orientale, Democratic Republic of the Congo, February 2011.

	Study population	
	(N= 3167)	
	n	%
Age (years)		
< 5	721	22.8
5-14	994	31.4
15-49	1183	37.3
≥ 50	269	8.5
Gender		
Male	1530	48.3
Female	1637	51.7
	Households	
	(N=552)	
	n	%
Household size (total)		
1 member	21	3.8
2-4 members	208	37.8
5-7 members	184	33.3
8-10 members	93	16.8
≥ 11 members	46	8.3
Mean household size (members)	5.7	
Minimum, maximum (members)	1,23	
	Heads of households	
	(N=552)	
	n	%
Age (years)		
Mean, median	39.9, 38	
Minimum, maximum	17, 86	
Gender		
Male	275	49.8
Female	277	50.2
Civil status		
Married	393	71.2
Single	62	11.2
Widow	50	9.1
Divorced, separated	47	8.5
Illiteracy		
All	138 / 552	25.0
Male	20 / 275	7.3*
Female	118 / 277	42.6*

* p<0.001

### Socio-economic conditions

Socio-economic conditions of the study population were homogenous both within the two different health zones and between the two health zones. See Table 2 for a general overview on socio-economic conditions of the population surveyed.

**Table 2:** Overview on socio-economic conditions of the study population interviewed in Aba and *Faradje health* zones, Province Orientale, Democratic Republic of the Congo, February 2011.

	Households	
	(N=552)	
	n	%
Level of lodging		
Traditional hut	527	95.5
House constructed with bricks / adobes	24	4.3
No house (burned down)	1	0.2
Type of latrine		
Simple pit latrine	476	86.2
No latrine	72	13.1
Cemented latrine	4	0.7
Drinking water sources		
Non-rehabilitated spring / River, lake	469	84.9
Rehabilitated spring	83	15.1
Fuel for cooking		
Firewood collected	537	97.3
Charcoal from vendors	15	2.7
Agricultural property		
Farmland less than 2.000 m^2^ (subsistence farming)	313	56.7
No farmland	123	22.3
Farmland more than 2.000 m^2^	116	21.0
Household assets		
Radio	232 / 552	42.0
Bed	130 / 552	23.6
Chicken	285 / 552	51.6
Sheep	244 / 552	44.2
Pig	30 / 552	5.4
Cow	4 / 552	0.7
Means of Transportation		
No means of transportation	249 / 552	45.1
Bicycle	292 / 552	52.9
Motorcycle	46 / 552	8.3

### Income, expenditures, actual problems and self-perceived living situation in the study households

In 46.7% (258/552) of the study households the amount of the weekly income of all working household members was estimated at less than US$ 5.5, in 33.3% (184/552) it was between US$ 5.5-11 and in 20.0% (110/552) it was more than US$ 11 (Exchange rate was US$ 1 = CF (Congolese Franc) 910 at the time of the study in March 2011). No difference could be seen in the estimated incomes of the population in the two health zones.

To make a living, 57.2% (457/799) of the working study population worked as farmers, 22.7% (181/799) as street hawkers (mainly selling local drinks and local products) and 13.4% (107/799) as craftsmen. All other activities made less than 2%.

The major expenditure in the study households was for food (58.0%, 320/552), followed by health care (25.4%, 140/552) and school fees (9.4%, 52/552). All further expenditures were mentioned in less than 2% of the households.

As main actual problem the households cited the lack of sources of income (42.9%, 237/552), violence and/or insecurity (25.4%, 140/552) and lack of access to health care (20.5%, 113/552). All further problems were mentioned in less than 2% of the households. Nine households (1.6%) did not mention an actual problem.

The self-perceived current living situation was in 40.4% (223/552) of the households fairly good to good, in 22.5% (124/552) fairly bad and in 37.1% (205/552) bad. 

### Estimation of maximum amount of user fees for health care households would be able to contribute

Of all interviewed study households 92.2% (509/552) estimated that they would be able to contribute to consultation fees. Of those, the median maximum amount of user fees for consultations would be US$ 0.27. Of all interviewed study households 78.6% (434/552) estimated that they would be able to contribute to the costs for drugs. Of those, the median maximum amount of user fees for drugs would be US$ 1.10. Six percent (33/552) of the interviewed households opted for free consultation and 18.8% (104/552) for free drugs (Table 3).

**Table 3:** Maximum amounts of user fees for consultation and for drugs the interviewed study households would be able to contribute in Aba and Faradje health zones, Province Orientale, Democratic Republic of the Congo, February 2011.

	Study households	Aba health zone study households	Faradje health zone study households
	N=552	N=336	N=216
Consultation fees			
	n / [%]	n / [%]	n / [%]
Households able to contribute	509 / 92.2	313 / 93.2	196 / 90.7
Households for free consultations	33 / 6.0	15 / 4.5	18 / 8.4
Households without answer^1^	10 / 1.8	8 / 2.3	2 / 0.9
Maximum consultation fees	N=509	N=313	N=216
Median [US$]^2^	0.27	0.22^3^	0.44^3^
Mean [US$]	0.50	0.36	0.72
Q_25_-Q_75_ [US$]	0.11-0.55	0.11-0.55	0.22-0.82
Minimum, Maximum [US$]	0.05, 5.49	0.05, 2.75	0.05, 5.49
Costs for drugs			
	n / [%]	n / [%]	n / [%]
Households able to contribute	434 / 78.6	245 / 72.9	189 / 87.5
Households for free consultations	104 / 18.8	81 / 24.1	23 / 10.6
Households without answer	14 / 2.6	10 / 3.0	4 / 1.9
Maximum costs for drugs			
Median [US$]	1.10	1.10^3^	2.20^3^
Mean [US$]	2.17	1.47	3.07
Q_25_-Q_75_ [US$]	0.55-2.75	0.55-2.20	0.99-3.30
Minimum, Maximum [US$]	0.05, 32.97	0.11, 21.98	0.05, 32.97

1 Although the interviewers carefully explained and re-explained the purpose of this question for at least 10 minutes, the households were not able to provide an answer.

2 Exchange rate was US$ 1 = CF (Congolese Franc) 910 at the time of the study (01 03 2011)

3 Significantly different between Aba and Faradje health zones (p<0.001)

### Health seeking behaviour in the study population

In 524 (94.9%) of the 552 study households at least one household member had fallen sick between January 1, 2011 and February 13, 2011 (day prior to the start date of the field work).

Seventy-one (13.5%) of the last household members being sick prior to the study did not seek health care due to lack of money. 

Of the 77.9% (408/524) patients who did seek care, 42.1% (172/408) went to a public health structure (communal or governmental), 24.3% (99/408) went to a private and 33.6% (137/408) to a faith-based structure. Public health structures were for free as the costs were covered by ECHO via MI. The median costs in a private health facility were US$ 3.98 and in a faith-based structure US$ 6.87. 

## Discussion

Our study was the first to estimate the socio-economic conditions of the population in the very remote health zones of Aba and Faradje in north-eastern Congo, and to assess their ability to contribute to health care.

General characteristics of the study population and their socio-economic conditions were very homogenous. No differences were noted within and between the two health zones. Living conditions were very basic, with almost the entire study population living in a traditional hut, cooking with firewood and fetching water from a non-rehabilitated spring. It has to be noted that almost 13% of the study population did not have any latrine. In the Multidimensional Poverty Index that Alkire and Santos introduced in 2010 the ‘standard of living’ was a new indicator to assess the deprivations that trap people in poverty, a fact that could be seen in our study [36].

The major expenditures in the study households were for food, health care and school fees.

As two-third of the study population works as subsistence farmers and the study area is very rural, we would have expected fewer expenses for food. Subsistence farming can play an important role in reducing the vulnerability of rural food-insecure households as it helps to mitigate high food price inflation [37,38]. Obviously in the study population the intermittent disruptions of agricultural activities have disturbed a production already at its limits, thus leading to scarcity.

Expenditures for health care are related to the actual health system in DRC, which is completely customer financed. Although MI is providing free health care, the health care is only provided in public health facilities; more than half of the study population, however, went to private or faith-based health structures that both are charging fees for consultation and drugs. This survey did not enquire about reasons for choosing a particular health facility, however other studies already have documented that private health facilities are the preferred sources of care for various reasons, such as closeness to the living places and the believe that quality of care is inevitably linked to high costs [39,40,41]. In one study this habit was particularity seen among the rural poor [42]. Informal statements given by the study population to the main author in person while conducting the field work of the study confirmed these believe. 

As government contribution is non-existing, pupils have to pay school fees at all school levels; monthly fees for primary schools are around US$ 1.1, for secondary schools US$ 2.7-3.8 (personal communication by the interviewers, DRC, 2011). As an average study household has one to two members of school age, school fees are a major burden for households. This is a common fact especially in low-income countries such as DRC and already widely discussed elsewhere [43,44].

The estimated income of the study population was low, even according to the revised absolute poverty level threshold of US$ 1.25/person/day defined by the World Bank [45,46]. With an average of 6 members per household, more than 80% of the households lived below the absolute poverty line. Only 20% of the study population had US$ 1.5 per day. Accordingly, the lack of sufficient sources of income was mentioned as the main actual problem. Still, almost half of the study population perceived their current living situation as fairly good to good.

When asked about the ability to contribute to health care services, more than 90% of the study population estimated to be able to do so for consultation fees and around 80% for costs for drugs. This level was beyond our hypothesis that 75% of the population would be able to contribute to health care.

Differences were seen in the estimation of the maximum amount to contribute to consultation fees -US$ 0.27- and costs for drugs -US$ 1.10. It is remarkable that people are more willing to contribute for drugs than for the consultation. In DRC costs for health care are usually split into consultation fees and costs for drugs, which seems to be relatively unique as in the few studies carried out in comparable settings in Africa no distinction between these two kinds of costs was made [47].

These results make us believe that the study population is able to contribute to a certain amount to health care. Moreover, the fact that more than half of the patients seek health care in rather expensive private or faith-based structures, underlines our assumption that part of the population can contribute to some extent to health care.

Nevertheless, we are well aware that being able to pay for health care does not automatically mean that the population ought to pay such fees. It should not be taken for granted that people in developing countries contribute to the costs of health care from their own resources. We are as well aware that out-of-pocket payments for health care are barriers to access. There are differences in health-care utilization rates related to the ability to pay (i. e. [48,49,50,51]). To some extent this could be seen in our study as well. Around 13% of the study population did not seek care while being sick due to related costs. When asked what to contribute to health care 6% opted for free services and 18% for free drugs. A number of smaller surveys carried out elsewhere in DRC have found that 10-20% of people requiring care do not receive treatment due to financial reasons [11]. A study in Tanzania revealed that the very poor might not be able to pay for health services even when the quality of services is improved [52]. An effective system of exemptions and waivers will be required for the very poor who are not able to pay for health services. 

Two similar studies were conducted in South-Kivu Province in DRC in 2009 [53,54]. In terms of major expenditures the results were similar to our study, however priorities were different. Expenditures for health care were only mentioned in fourth place, whereas in our region they came second. Moreover, the price both for consultation and for drugs considered feasible to contribute by the population was twice as high as in our study. On the other hand the estimated income was as well twice as much compared to our target population. It seems thus probable that the population is able to contribute to health care with higher prices, as they seem better off in general.

In our literature research we did not find other studies regarding population’s estimations about their ability to contribute to health care, neither in DRC nor in other developing countries although this subject is of importance [15].

Some data and methodological limitations of our study should be mentioned. Two days before starting data collection in the field the security situation in Faradje town after an LRA attack did not allow the two quarters selected there to be visited. As all study results were very homogenous (design effect around 1), omitting 48 households did not hamper the statistical validity of our results, as 288 households would have been enough to have statistical power. 

Accessibility to the study population was a challenge as infrastructure in the region is almost non-existing; most of the roads are not even passable by 4-wheel-cars. In order not to limit our cluster selection to villages accessible by car, we had half of the interviewers moving on motorcycles or on foot to reach all villages randomly selected prior to the study. The different means of transport further allowed us to spend enough time in all villages and with all heads of households in order to have reliable answers.

Lastly, population estimates were based on the most recent population census carried out on 2003 and extrapolated with an annual growth rate of 3%. We are aware that there has been substantial population movement from 2008 due to the security situation with massive displacement and ongoing return. Still these were the only figures available.

As a result and following this study, MI in April 2011 suggested introducing flat-rates for health care services. As it was obvious that the population was more willing to contribute to drugs than to consultations, we proposed a dual system with separate contributions for drugs and consultations. The contribution to consultation fees was US$ 0.16 and for drugs US$ 0.34. These two cut-off points were chosen in accordance with the limits of the lower quartile mentioned by the study population while estimating the maximum amount of user fees. Therefore we are confident that at least three-quarter of the population will be able to contribute to health care services. For the 10% of the population who will not be able to contribute to health care services, free access is assured through so-called equity funds to be administered according to health committee recommendations.

In conclusion it has to be said that once the project ends, the population will have to pay again full price for their health service. One of the better solutions would be the introduction of a health care financing system with the goal to reach universal coverage to health care. Another possibility is to reduce consultation fees and drug prices together with a quality increase under external assistance programs as it obvious that utilization of health care services will then increase significantly. However political realities in the DRC oblige us to prepare the population again for a health system without external support or any health care financing system.
